# Improving Physical Activity Resource Guides to Bridge the Divide Between the Clinic and the Community

**Published:** 2008-12-15

**Authors:** Hilary K. Seligman, Melanie D. Grossman, Nathalie Bera, Anita L. Stewart

**Affiliations:** Division of General Internal Medicine, San Francisco General Hospital; University of California, San Francisco, California; University of California, San Francisco, California; University of California, San Francisco, California

## Abstract

**Introduction:**

Primary care providers have limited time for physical activity counseling. They can optimize counseling time by referring patients to community resources for more comprehensive support. To facilitate referrals, resource guides (lists of community opportunities with descriptive information) are often created but seldom used. We elicited the detailed opinions of providers about how to make resource guides more useful for them.

**Methods:**

We asked a convenience sample of health care providers open-ended questions about resource guide usefulness. Providers included 7 physicians, 6 physical/occupational therapists, 5 registered nurses, and 2 nurse practitioners practicing in diverse settings. We identified key themes using grounded theory methodology.

**Results:**

All participants thought resource guides were potentially useful, particularly providers who worked in communities that were socioeconomically or culturally different from their own. Perceived benefits included providing easy access to information, facilitating specific activity recommendations, and reminding health care providers about the scope of available opportunities. Participants cautioned that resource guides were not a substitute for individual recommendations or provider counseling. They said resource guide usefulness was limited by inconvenience, frustration with outdated entries, and discomfort referring patients to programs without personal experience of program quality. Providers offered suggestions for useful information to include in the resource guides.

**Conclusion:**

Resource guides may offer a critical link between clinical services and community resources. Integrating guides with existing clinical systems, incorporating mechanisms for frequent updating, and providing multiple copies will help address provider concerns. Web-based resource guides may help achieve these goals.

## Introduction

Regular physical activity helps prevent and manage multiple chronic diseases, including cardiovascular disease, hypertension, type 2 diabetes, and obesity. Among older adults, regular physical activity also reduces functional limitations and medication dependence, prolongs independent living, and improves quality of life (1,2). It is thus appropriate that counseling to increase physical activity occurs in the clinical setting (2,3). Patients expect their health care providers to offer such information, and providers generally accept and value their role in promoting physical activity (4). However, the brief, low-intensity physical activity counseling that is generally offered by providers in the clinical setting has limited effectiveness (5).

Many experts believe that broad-scale provision of effective physical activity counseling in the clinical setting is unfeasible. A more effective strategy, these experts contend, may include the use of limited available clinical time to refer patients to community resources that are able to provide more comprehensive support for behavioral change (5-10). For example, a central element of Wagner's chronic care model is that health care organizations develop partnerships with, and providers encourage patient participation in, community programs (9). Consequently, many departments of health and community agencies have invested substantial time and effort in developing resource guides, with the goal of increasing provider awareness of the breadth and variety of existing community resources. These resource guides, designed for providers and patients, provide contact and/or descriptive information for community resources. Samples of resource guide formats are available on the Internet and in peer-reviewed literature (11,12).

Several studies have successfully incorporated resource guides into multicomponent interventions designed to increase physical activity levels (9,13,14). Studies that attempt to isolate the effect of resource guides have demonstrated increases in provider counseling about exercise, referrals to community resources, and patient physical activity levels (11,15,16). Studies of other types of resource guides have shown they may also improve the match between the services providers desire for their patients and the services to which the providers refer (17).

Health care providers have expressed favorable attitudes about referring patients to community resources for physical activity, such as fitness professionals and fitness centers (15,18). Physical activity instructors often indicate a need for more program participants and a desire for more referrals from health care providers (19). Despite these common interests, few referrals are actually made from the clinical setting to community resources (17,18), and resource guides provided to health care providers are infrequently used (16,17,20).

Prior studies have sought recommendations from community members about how to improve the usefulness of resource guides (11,21). However, we are not aware of any studies that solicited the detailed opinions of health care providers about how to improve the usefulness of resource guides. We conducted formative research by soliciting the opinions of health care providers about their experience with resource guides and how resource guides might be made more useful in the clinical setting. Although we specifically addressed physical activity resource guides, much of our data is also applicable to the development of other types of resource guides.

## Methods

The Community Healthy Activities Model Program for Seniors (CHAMPS) is a community-based public health program that increases physical activity levels among older adults (22,23). As an initial step toward disseminating CHAMPS into low-income and minority communities, researchers at the University of California, San Francisco (UCSF), with the support of 2 local public health departments (City of Berkeley Division of Public Health and the San Mateo County Health Services Agency), discussed elements of the CHAMPS program with a wide-ranging group of key stakeholders (19). The data for this analysis come from those in-depth interviews with health care providers.

We interviewed 20 health care providers, including registered nurses, physical therapists, occupational therapists, nurse practitioners, and physicians in the public and private sectors. Providers were a convenience sample identified by UCSF, the partner health departments, and their contacts with local hospitals and clinics as providing care to low-income or multicultural patients. To understand how links from the clinical setting to the community were being made and how they might be improved, we asked providers the following questions:

- "What resources or tools do you have or use now to support your own efforts in physical activity assessment or counseling for older adults?"
- "Would it be helpful for you to be able to give to your older patients a resource guide that lists all the physical activity programs and classes available for older adults in your county/city?"
- "Would someone need to review the classes [before entry into the resource guide] for quality of instruction, etc.?"

The interviewer asked follow-up questions to clarify specific points. At other times during the interview, we asked providers to comment on *Healthy People 2010* goals for physical activity, counseling habits, comfort and confidence with counseling, barriers to counseling, recommendations for medically clearing patients before recommending enrollment in community-based programs, and other resources or tools desired to support efforts in assessing and counseling about physical activity. When participants shared thoughts about resource guides in the context of these other topics, we included the data in this analysis. A physician trained in preventive medicine (N.B.) conducted all interviews. Interviews lasted approximately 1 hour and took place between July and December of 2002. We offered reimbursements of $100 to physicians and $50 to other health care providers.

Interviews were audiotaped and transcribed verbatim. We used a grounded theory approach to systematically identify all dimensions of comments about resource guides. One author (H.S.) initially read each transcript to identify themes emerging from the text and incorporated themes into a coding scheme. Transcripts were reanalyzed by 2 of the 3 reviewers (H.S., N.B., and M.G.); each reviewer independently assigned text units to identified themes. During consensus meetings, we added new themes to the coding structure as they emerged, identified and resolved coding discrepancies, and discussed properties of each theme. We used NVivo software version 7.0 (QSR International, Victoria, Australia) to facilitate the analysis.

## Results

### Provider characteristics

We solicited the opinions of 20 health care providers during 18 interviews. (For convenience, in 2 interviews we interviewed 2 providers who had different occupations but worked in the same setting.) Providers worked in public and private settings and in home, outpatient, and inpatient settings (Table 1). Providers also drew on their experiences in former jobs. The patient panels of the interviewed providers varied socioeconomically, racially, and by functional status and medical comorbidities. For example, 1 private sector nurse worked with predominantly lower socioeconomic status African Americans who had multiple medical comorbidities, while another private sector nurse worked with predominantly middle- and upper-class Filipino and white patients who had had a myocardial infarction but generally had few other medical comorbidities.

### Perceived usefulness of resource guides

Of the 20 providers, 3 already had physical activity resource guides available to them, 13 had no resources that cataloged existing community resources, 3 had resource guides that were too limited to be useful, and 1 generally referred patients to a social worker if a community referral was appropriate. With the exception of those working with very frail elderly patients, all providers thought community resources for physical activity were important for helping patients become more physically active: "What would be most helpful to me would be to have the availability of more walking clubs, or support groups, or aerobics classes within walking distance" (physician, public sector). All participants also thought that physical activity resource guides were a potentially useful adjunct to their counseling, again with the exception of those who worked with frail older adults. These providers thought resource guides would be of limited use because "some [patients are] just are not capable of doing that kind of thing on their own" (physical therapist, public sector).

Providers who lived in the same communities as their patients generally had greater knowledge of available resources than those who lived in different communities. Providers who lived in different communities were often those who served vulnerable populations. One public sector physician stated, "I don't live in the area that I practice, so I can't ad lib as much. If I were still working here in the city, I could say 'Well, you know, there's the Y here, there's the Y there.' " Another private sector physician expressed similar concerns:

. . . especially if you're not from this community. I don't live here. Not that I live that far, but I literally do not live here, and so I have no idea [where appropriate resources for my patients are]. I mean, it's kind of embarrassing, but I actually don't.

Similarly, providers who were of a different cultural or ethnic background as their patients expressed limited knowledge of appropriate resources:

I realize today that I actually don't know about the community programs available to my patients in this area. Some of them being Hispanic, I don't know about any sort of a senior program that might be appropriate for them. (physician, private sector)

One public sector physician explicitly voiced the mismatch between known resources and culturally appropriate resources in emphasizing the importance of finding relevant opportunities: "So what you say to 1 person who maybe has no car, no financial stability at all, new immigrant, undocumented — you don't tell them to run out and join 24-Hour Fitness [a national chain of gyms available for a sign-up fee and monthly dues]."

### Benefits of resource guides

All participants thought resource guides offered potential benefits. These benefits fell into 3 general areas: providing easy access to information not otherwise readily available, facilitating more specific activity recommendations, and reminding providers of the scope of available services.

Providers who had no access to resource guides often were not able to refer patients to community opportunities because of a lack of time or personnel necessary to obtain information: "And the reality is it's difficult to get this [information] without having the manpower to say, 'Can you find out for me about this?' " (physician, private sector). This caused frustration for some providers:

And then, today I went to my colleague and I said, "What is the name of that thing that they do at [the mall]?" I didn't even know, or who I would call. We actually have someone here who does community health. But I don't have her number. What other tools do I have? No other real resource or tools. (physician, private sector)

In the absence of a single information source, 1 provider based her recommendations on "what 1 patient told me a few weeks ago. I asked because it's the only way I find out about things" (physician, public sector). Another provider thought a resource guide would be particularly advantageous for her because of the importance of advice from "the person they trust" (registered nurse, private sector).

Three participants thought the primary advantage to resource guides was in providing specifics about available resources. These participants thought that their counseling was more effective if it was more specific: "If you're given something exact to do, [you're] actually more willing to do it than to [just] say 'Exercise!' " (physician, private sector). These providers thought the advantage to offering information that is more specific was in not "overwhelming" their patients:

I think we really need to have the resources and phone numbers, the contact person. Versus, "There's a program down at X, you ought to check it out," kind of thing — it's not going to happen. (registered nurse, private sector)

Three participants indicated that they sometimes forgot to talk with patients about available community resources, even when they knew about them: "We actually have, right down here at our community center, an aerobics class, and also an exercise class for seniors, that I know I forget to refer people to" (physician, public sector). A resource guide might help remind providers about available resources and encourage referrals when they might not otherwise occur:

If I knew that there were a program, and knew more about it, and knew what the skill of the person running the program was, then there may be patients that I could refer to that kind of a setting. (physical therapist, private sector)

Other participants thought that by giving providers more information, resource guides would also be giving patients more choices in activities to pursue.

### Concerns about resource guides

Providers voiced a number of concerns about the usefulness of resource guides. Two, a registered nurse and a nurse practitioner, emphasized that resource guides would not alleviate the need to counsel their patients about physical activity resources: "Just giving it to them in their hand, and not taking them further — that's a problem" (nurse practitioner, public sector). Some participants also thought that resource guides might reduce the burden of looking up available community resources, but not eliminate it unless the guide were "readily available, so they're easy to grab at hand" (physician, public sector). Interviewed providers differed in the ways in which they used resource guides in the clinical setting. Some providers spoke of photocopying lists of resources and handing them to patients, and others referred to consulting a resource guide and then discussing available opportunities with patients verbally. To improve efficiency, a registered nurse in the private sector suggested that guides covering many different domains be joined into a single book, because "it's hard enough trying to keep track of everything in one's life, without having to look at 3 or 4 different books."

Four participants cited as a concern the necessity that resource guides be updated frequently in order to remain useful. A public sector physician shared her experience with resource guides in her clinic: "We get a guide, and then 4 years later, we're still handing out the same thing, and half the things aren't on it anymore, or they're horrible."

Four providers mentioned that they had more "confidence" referring to programs or facilities of which they had first-hand knowledge, rather than just a listing in a resource guide. A private sector physical therapist said, "I would feel more comfortable knowing what I'm actually referring to." One of these providers suggested that resource guides be supplemented with visits to their practices from staff members at activity sites. Alternatively, "I also would appreciate time to go there myself, just so I could see what I'm getting these people into" (physical therapist, public sector).

### Helpful information to include in physical activity resource guides

All participants thought that having someone review community resources for quality before entry into the guide would be helpful, although 2 doubted how helpful such a review might actually be:

We have a list of community-based exercise programs for our patients. We have reviewed most of them, and yet saying that, I'll also say in the next breath that it's sort of irrelevant because it is teacher-dependent, and the teachers often switch. (registered nurse, private sector)

Table 2 lists other categories of information participants thought would be helpful to include in a physical activity resource guide. Except for clarifications, participants freely offered these categories without direct prompting by the interviewer.

## Discussion

Health care providers find resource guides to be a potentially useful adjunct to physical activity counseling. More ready access to information about community resources allows providers to supply specific information to their patients that they might not otherwise be able to supply. However, several concerns limit the usefulness of resource guides. Providers say that resource guides cannot act as a substitute for individual counseling or be relied on to accurately communicate the quality of community programs. They also think resource guides should be convenient to access and frequently updated.

These concerns suggest a number of concrete strategies that might make resource guides more useful. These strategies include 1) requiring that listed programs frequently update their entries to maintain their listing, 2) regularly redistributing updated resource guides, 3) providing clinical practices with enough copies for distribution into each exam room or with each practitioner, and 4) pooling resources with health departments or other agencies creating other types of resource guides (such as resources for elderly or homeless patients) to provide a single, comprehensive book of local services. The latter recommendation stems from participants' concerns about keeping track of multiple resource guides ("3 or 4 different books") addressing the needs of different populations. Web-based resource guides may achieve many of these goals, particularly in health systems with electronic medical records well integrated with existing clinical flow. Insofar as resource guides act as a reminder about the scope of available services, they might also include activity opportunities not traditionally considered for resource guides, such as malls, school tracks, public parks, and other safe places to walk; local listings for instructional programs on television, such as the *Sit and Be Fit* program available on Public Broadcasting Service stations; and recommendations for suitable videos. These strategies each require that additional resources be invested in the creation and dissemination of resource guides, but these improvements will make them considerably more useful for health care providers.

When providers offered suggestions for important content areas to be included in a resource guide, many areas emerged, without clear consensus on the most important ones. Had we asked participants directly about which content areas were most critical for inclusion, it is possible that some consensus would have emerged. The lack of consensus, however, suggests that resource guides might need considerable detail if they are to be useful to a range of provider types.

Resource guide developers should consider how the format of their guide influences the ways in which it is used and target guides toward their intended users. Resource guides intended to be given to patients, for example, should be written and formatted to facilitate understanding for all literacy levels.

We found that providers who were least familiar with referral opportunities tended to be those who were most different — socioeconomically or culturally — from their patients. Limited knowledge of available community resources may decrease provider confidence in their ability to be an effective counselor, which in turn may decrease provider counseling rates (27). Thus, reduced awareness of community resources appropriate for low-income and ethnically diverse patients may partially explain socioeconomic and ethnic inequalities in counseling rates (24-26). Lack of awareness of referral opportunities may also contribute to the pessimism with which providers in the public sector view their ability to be effective physical activity counselors (28). How knowledge of local community resources affects counseling strategies and counseling rates deserves further research.

Many providers noted that physical activity resource guides were helpful because providing patients with specific activity suggestions was more effective than providing general advice in facilitating behavior change. The literature generally supports this assertion (26,29). Many health communication experts recommend that providers increase the specificity of the information communicated to patients to enhance recall and facilitate behavior change (30-32). This strategy may be particularly important for older adults (33). Resource guides might also offer providers the necessary information to make physical activity recommendations tailored to individual patient barriers, an approach to counseling supported by the transtheoretical model (34). For example, patients who report difficulty walking may be referred to community pools, or patients who report feeling unsafe walking outside after dark may be referred to local malls.

Our study has several strengths. To our knowledge, it is the first study that solicits the detailed opinions of diverse health care providers about their experiences with resource guides. The use of open-ended questions and qualitative data analysis allowed us to identify a broad range of potential benefits and concerns that we may not have discerned in a closed-ended survey.

Our study also has several limitations. Because we conducted the interviews to enhance the dissemination of a broad-based physical activity program, we did not specifically ask providers how to design better resource guides to meet their needs. Second, our sample was limited to providers who practiced in northern California, an area rich in community resources for physical activity; these resources may increase the role for resource guides compared with areas that are resource-poor. Third, we drew our sample to represent a broad range of provider backgrounds and practice settings. Although this sampling strategy allows us a diversity of opinion, the small number of providers of each type limits our ability to understand how resource guides may be used differentially in different practice settings. Fourth, because our study concentrated on providers' needs with respect to their older patients, the opinions expressed may not fully represent how to make physical activity resource guides more useful for providers who take care of younger patients. Finally, for this topic area we did not reach thematic saturation, and thus additional perspectives likely exist on the use of resource guides in the clinical setting that we did not obtain.

Clear evidence suggests that creating or enhancing access to places for physical activity combined with informational outreach activities is effective at increasing physical activity levels across diverse settings and populations (35). With few minutes available for physical activity counseling during the clinical encounter, health care providers must find strategies for linking patients with services that provide more comprehensive support. Resource guides may offer this critical link. However, integrating resource guides into existing clinical systems and creating features that meet the needs of health care providers are necessary if such guides are to reach their full potential. Resource guides may be important adjuncts to counseling for providers working in low-income and culturally diverse communities, where providers may have less knowledge of available community resources.

## Acknowledgments

This research was supported by grant no. 044223 from the Robert Wood Johnson Foundation.

## Figures and Tables

**Table 1:** Characteristics of Health Care Providers (N = 20) Interviewed About Physical Activity Resource Guides, by Occupation, Setting, and Sex, Northern California, 2002

Occupation of Participant	Occupational Setting Represented by Interview^a^	Female (n)
Physician^b^ (n = 7)	Public community clinics (n = 2)	5
	Private hospitals (n = 2)	
	Public geriatrics clinic	
Registered nurse^b^ (n = 5)	Private home care program	5
	Public community clinic	
	Private hospital, cardiac rehabilitation program	
	Public geriatrics clinic	
	Private hospital	
Physical therapist^b^ (n = 4)	Private home care programs (n = 2)	3
	Public hospital	
	Clinic in public adult day center	
Occupational therapist (n = 2)	Public hospital	2
	Clinic in public adult day center	
Nurse practitioner (n = 2)	Private hospital	2
	Clinic in public adult day center	

a Some participants worked in more than 1 occupational setting.

b Two interviews had 2 participants (a private sector nurse and physical therapist in 1, and a public sector nurse and physician in the other).

**Table 2:** Information to Include in Physical Activity Resource Guides, Suggested by Health Care Providers (N = 20), Northern California, 2002^a^

**Domain**	No. of Participants Mentioning the Domain
Level or types of activities offered (including appropriateness for older adults)	8
Accessibility by public transportation	5
Cost	4
Leader requirements and certification	4
Hours of operation	3
Languages spoken or cultures to which activities are tailored	3
Wheelchair accessibility	3
Class size	2
Locations of parks and public spaces	1

a Opinions of 20 health care providers interviewed about how to make physical activity resource guides useful for their practice.
